# Artificial Intelligence and Radiomics for Endometrial Cancer MRI: Exploring the Whats, Whys and Hows

**DOI:** 10.3390/jcm13010226

**Published:** 2023-12-30

**Authors:** Elisabetta Leo, Arnaldo Stanzione, Mariaelena Miele, Renato Cuocolo, Giacomo Sica, Mariano Scaglione, Luigi Camera, Simone Maurea, Pier Paolo Mainenti

**Affiliations:** 1 Department of Advanced Biomedical Sciences, University of Naples “Federico II”, 80131 Naples, Italy; 2 Department of Medicine, Surgery and Dentistry, University of Salerno, 84081 Baronissi, Italy; 3 Department of Radiology, Monaldi Hospital, Azienda Ospedaliera dei Colli, 80131 Naples, Italy; 4 Department of Medicine, Surgery and Pharmacy, University of Sassari, 07100 Sassari, Italy; 5 Institute of Biostructures and Bioimaging of the National Council of Research (CNR), 80131 Naples, Italy

**Keywords:** radiomics, artificial intelligence, MRI, endometrial cancer

## Abstract

Endometrial cancer (EC) is intricately linked to obesity and diabetes, which are widespread risk factors. Medical imaging, especially magnetic resonance imaging (MRI), plays a major role in EC assessment, particularly for disease staging. However, the diagnostic performance of MRI exhibits variability in the detection of clinically relevant prognostic factors (e.g., deep myometrial invasion and metastatic lymph nodes assessment). To address these challenges and enhance the value of MRI, radiomics and artificial intelligence (AI) algorithms emerge as promising tools with a potential to impact EC risk assessment, treatment planning, and prognosis prediction. These advanced post-processing techniques allow us to quantitatively analyse medical images, providing novel insights into cancer characteristics beyond conventional qualitative image evaluation. However, despite the growing interest and research efforts, the integration of radiomics and AI to EC management is still far from clinical practice and represents a possible perspective rather than an actual reality. This review focuses on the state of radiomics and AI in EC MRI, emphasizing risk stratification and prognostic factor prediction, aiming to illuminate potential advancements and address existing challenges in the field.

## 1. Introduction

Endometrial cancer (EC) is a prevalent gynaecological malignancy that affects thousands of women worldwide, with an estimate of 66,200 new cases and 13,030 new deaths for the year 2023 in the United States [1]. The remarkable clinical relevance of this neoplasm lies in its strong association with obesity and diabetes, risk factors that are common in the western world [2].

Traditionally, the management of EC has been based on surgical staging and histopathological analysis. However, recent advances in medical imaging, particularly magnetic resonance imaging (MRI), have revolutionized the field by enabling accurate risk stratification. Indeed, preoperative imaging modalities such as transvaginal ultrasound (TVS), computed tomography (CT), and magnetic resonance imaging (MRI) are valuable tools for assessing EC and lymph node involvement as well as for detecting distant metastatic disease. These imaging modalities help guide the optimal course of treatment by providing crucial information about tumour staging. In particular, CT and positron emission tomography/CT (PET/CT) have an important role in the evaluation of advanced EC for the assessment of disease outside the pelvis. On the other hand, MRI is highly effective for EC local staging and in identifying gross myometrial invasion and tumour extension to the cervical stroma, which can influence treatment decisions. Moreover, imaging is useful in monitoring treatment response and surveilling patients for early detection of recurrent disease [3]. Pelvic MRI has been widely recognized as a valuable imaging technique for preoperative staging of EC. It involves obtaining multiple T2-weighted sequences angled perpendicularly of the uterus, along with T1-weighted series with intravenous contrast, and DWI images with the corresponding ADC maps, the latter reflecting the tumour cellularity [4].

The reported sensitivities, specificities, and accuracies of contrast-enhanced MRI in detecting deep myometrial invasion, cervical stroma invasion, and metastatic lymph nodes vary widely based on studies published in the past decade [5,6,7], leaving the diagnostic performance of MRI with some extent of variability. Among the limitations of the MRI performance, the moderate sensitivity (43%) and specificity (73%) for detection of metastatic lymph nodes [8], as well as the significant variability in the interpretation of MRI findings among different operators and/or different sequences [7,9], have to be reported. Moreover, radiologists provide qualitative and semi-quantitative data from medical images to describe tumours and their heterogeneity, which may not effectively reflect the actual biological characteristics of the tumour [10]. Finally, the efforts made to increase standardization through imaging guidelines is undermined by their relatively limited adoption and diffusion [11].

To reduce these gaps, radiomics and artificial intelligence (AI) algorithms offer new avenues for risk assessment, early detection, treatment planning, and prognosis prediction in patients with EC. Radiomics, indeed, enables the conversion of images into quantitative data that can be analysed systematically, with the extraction of numerous quantitative parameters (referred to as radiomics features). These features capture the diversities within the images and offer valuable insights into the characteristics of the cancer phenotype [12].

The development and validation of robust radiomics models require large, diverse datasets and standardization protocols [13] and the road to integration of radiomics into clinical practice is still a long way. In the wake of this goal, this review aims to provide insights into the state of AI in EC MRI with a focus on risk stratification and prediction of prognostic factors, as well as shed light on potential advancements in order to address existing challenges.

## 2. Prognostic Factors Related to Tumour Stage

MRI is highly recognized as the most valuable imaging technique for local staging and as such, current guidelines recommend its routine use in the pre-treatment workup of patients affected by EC [14]. In 2019, imaging guidelines regarding MRI acquisition protocol and image interpretation in this setting have been released by the European Society of Urogenital Radiology, with the objective of standardizing and optimizing the procedure [15]. The most recently updated and adopted staging system from the International Federation of Gynecology and Obstetrics (FIGO) includes data that cannot be assessed with MRI (e.g., molecular classification, histological type and grade); however, it is also strongly tied to the local tumour extent, as a matter of fact, and to major adverse prognostic factors detectable at MRI (including deep myometrial invasion—DMI—and the presence of lymph node metastasis—LNM) are also considered [16]. Thus, despite being a surgically staged disease, defining the most appropriate initial management relies on accurate and reliable MRI results [17]. Nevertheless, MRI still has some limitations in terms of diagnostic accuracy, with a certain degree of variability [18]. To further increase the value of MRI and address specific shortcomings in the evaluation of these tumour characteristics, radiomics and AI approaches have been recently investigated.

### 2.1. Deep Myometrial Invasion

The prognosis of EC hinges on various factors, including the stage of the disease, the extent of myometrial invasion, the presence of lymphatic vascular infiltration, histologic grade, and the status of lymph nodes [19]. Among these, DMI stands out as a critical prognostic indicator. DMI is defined as the infiltration of cancer cells into 50% or more of the myometrial wall thickness. It holds such significance that it is incorporated into the International Federation of Gynecology and Obstetrics (FIGO) staging system, which categorizes stage I EC into IA and IB [16,20]. This distinction is necessary because tumours with DMI have a higher likelihood of spreading to parametrial tissues and metastasizing to pelvic lymph nodes, indicating a more aggressive disease and influencing treatment decisions [21].

The assessment of DMI in cases of EC frequently yields differing results among general radiologists. The comparison between the initial reports (processed by general radiologists) and secondary reports (reinterpreted by radiologists specializing in gynaecologic oncologic imaging) made by Woo et al. interestingly showed higher levels of sensitivity and accuracy in the secondary reports, highlighting the additional value of specialistic review of MRI in EC patients made by operators with experience in gynaecologic oncology [22].

It is dutiful not to forget that several potential pitfalls may lead to errors in estimating myometrial invasion. These include difficulty in detecting isointense tumours on T2w images, reduced contrast at the endometrial–myometrial interface due to factors like fibroids or adenomyosis, and challenges in measuring thin myometrium in cornual regions, especially in older women with atrophic uteri and less discernible junctional zones and thinning of the endometrium due to age or distension of the endometrial cavity. In such cases, DWI and DCE imaging can aid in tumour detection, characterization, and evaluation of myometrial invasion [8]. 

Among others, there are four pilot studies deserving to be mentioned [23,24,25,26]. The one from Stanzione et al. proved the feasibility of a radiomics-powered ML model based on T2 images for DMI detection with an AUC of 0.92 and 0.94 in the cross-validation and final testing, respectively, but these highly encouraging metrics might be overly optimistic considering the very limited sample size [23]. Fasmer and colleagues had a bigger cohort and used contrast-enhanced T1w sequences for the segmentation of the whole tumour and resulted in a medium-to-high performance of radiomics signatures with an AUC of 0.84 and 0.76 in the training and validation cohort, respectively [24]; while interesting, the choice of using contrast-enhanced imaging for this radiomics task might limit the applicability of the model for patients in which contrast administration cannot be performed (e.g., kidney failure). Two studies from the same year investigated deep learning (DL) approaches, with Dong et al. being the first attempt to use AI technology to evaluate the DMI in early-stage EC; both studies agree that AI has the potential to serve as an auxiliary tool to radiologists [25,26].

With a study published in 2021, Han et al. explored a dual-sequence (T2w and DWI) radiomics approach employing whole-uterus segmentation and found that it did not have a significantly different diagnostic performance in predicting histological presence of DMI compared to conventional radiological assessment (the accuracy was 0.83 for the dual-sequence model vs. 0.84 for the visual evaluation) [27]. It should, however, be considered that the MRI reads were performed by two highly experienced radiologists working in consensus, which does not reflect common radiological practice and might have artificially boosted the performance [28]. Interestingly, they found that radiomics feature consistency was higher for the whole uterus compared to the lesion-based analysis (*p* < 0.05); this might be explained by the lesion segmentation being more dependent on the radiologist’s clinical experience compared to the segmentation of the entire uterus.

With a different approach, Zhao et al. compared a conventional diagnostic model (M1), a model of combined radiomics signatures from T2w and contrast-enhanced T1w images (M2), and a nomogram built with M1 and M2 (defined M3), with the latter reaching the highest diagnostic performance [29]. A more holistic approach appears desirable since it combines the strength of both quantitative as well as qualitative evaluation, and the study also has the merit of presenting an external validation. However, it has been highlighted that nomograms do not represent the most appropriate tool to handle radiomics models, as it will be further detailed later in the review [30].

More recently, Lefebvre and colleagues conducted a two-centre and retrospective study to establish standardized validation and ensure the reproducibility of computational methods. The authors extracted radiomics features from T2w, CE-T1w and DWI images, thus working with a comprehensive set of features from all the sequences currently included in the clinical MRI protocol. The author achieved a noteworthy and reliable performance, notably with an AUC of 0.81 for DMI in the test dataset. The consistency was maintained even in the presence of potential sources of variability, like differences in contouring and MRI image acquisition from various vendors, which would typically increase variability between data from different institutions [31].

Conversely, the radiomics model published by Miccò et al. suffered from a significant performance drop from the training to the validation set, with the diagnostic accuracy decreasing from 78% to 69% (AUC for training/validation of 0.85/0.68). These findings suggest a poor generalizability which might be due to the relatively low number of patients in the train set (*n* = 73) as well as the extended data collection period (2009–2019), in which MRI technology has certainly advanced, improving image quality and potentially influencing the extraction of textural radiomic features [32].

In line with the findings from the Han et al. study [27], Otani and colleagues found that an ML classifier did not outperform the performance of conventional MRI assessment [33]. Indeed, the AUCs of all radiologists for DMI detection (ranging from 0.83 to 0.88) were either higher than or equal to the mean AUC of the ML classifier (0.83). Interestingly, the authors observed limited and non-statistically significant changes in the radiologists’ performance when they were provided with the ML classifier predictions when performing the evaluation.

Finally, a multicentre 2023 study by Li et al. deserves to be mentioned [34]. With a large dataset (overall number of 495 EC patients, excluding EC stage II or higher) obtained from 15 different UK institutions and a dedicated test set, the authors aimed to build prediction models based on T2w whole-tumour radiomics features and clinical variables. Of note, no clinical variables were selected for the final DMI prediction model (build on a subset of the entire cohort) which showed a promising performance in the test set (AUC = 0.79). A brief synthesis of the main characteristics of scientific articles presented in this review regarding DMI can be found in Table 1.

### 2.2. Lymph Node Involvement

A precise and non-invasive assessment of LNM before surgery is essential. It offers valuable information for predicting prognosis and making treatment decisions, particularly when planning a lymphadenectomy. According to the FIGO guidelines, systematic lymphadenectomy is routinely carried out. Nevertheless, an ongoing debate persists regarding its necessity for patients with low-risk or stage IA disease [35], given the exceedingly low incidence of LNM in this group [36].

While MRI is rather valuable and reliable in the evaluation of EC local extent, its effectiveness in assessing LNM is limited, as reported sensitivities range from 25% to 50% [37,38]. Currently, LNM evaluation is exclusively based on a dimensional criterion (short axis values > 8 mm in pelvic nodes and >10 mm in para-aortic nodes are considered suggestive of LNM) [15]. Thus, in the last few years researchers have investigated the potential of radiomics to overcome MRI limitations in LNM assessment.

In 2019, Xu et al. tested a combined model to investigate the efficacy of MRI-based radiomics for preoperative prediction of metastasis in EC patients with lymph nodes not meeting the size criterion. The combined model, referred to as ModelCR1, incorporated radiomic features, lymph node size, and CA125 levels and demonstrated superior discriminative capabilities, especially in patients with not-enlarged lymph nodes, significantly improving sensitivity compared to standard MRI reports [39]. Fasmer et al. compared radiomics signatures derived from whole-tumour segmentation and single-slice segmentation and it was observed that both signatures showed comparable performance metrics in the training group. However, in the validation group, the whole-tumour signatures demonstrated superior performance in predicting LNM (whole-tumour AUC in train/test sets of 0.73/0.72 vs. single-slice AUC in train/test sets of 0.83/0.56 for LNM). The findings suggest that employing whole-tumour radiomic profiling in EC could offer advantages as it incorporates data from the entire tumour, potentially leading to improved predictive accuracy [24]. More recently, Otani et al. assessed the effectiveness of ML classifiers combined with clinical features (including tumour markers) in comprehensively evaluating pre-treatment risk factors, such as pelvic and para-aortic lymph nodes (PLNM and PALNM), reaching a comparable AUC of 0.72/0.82 for PLNM/PALNM [33]. These authors converge on the result that radiomics can assist radiologists in enhancing their ability to predict PLNM metastasis in EC cases, particularly in reducing false positive diagnoses (nodes > 8 mm) and false negatives (nodes ≤ 8 mm). Liu et al. focused their research on early-stage EC patients (i.e., stage I), with the objective to develop a predictive model for the identification of those who would benefit from a lymphadenectomy [40]. They built a nomogram integrating a radiomics signature along with two preoperative clinical risk factors (CA125 levels and MRI-detected DMI), which demonstrated strong predictive capabilities for LNM in both the training and test groups (AUC in train/test set of 0.85/0.83), outperforming the clinical model applied in the clinical routine at the same institution to decide whether a lymphadenectomy should be performed. However, the radiomics signature required the manual segmentation of all the MRI sequences included in the clinical protocol. Similar findings have been reported by Bo and colleagues, with a nomogram developed integrating MRI radiomics and non-radiomics features of the EC lesion as well as clinical parameters like CA125 exhibiting a strong predictive performance for PLNM [41]. These results are also in line with those from a different research group, which has also the merit of highlighting the notable advantage in the use of a computer-assisted segmentation technique [42]. Indeed, this could mitigate the potential biases associated with the selection of small pelvic lymph nodes in MR imaging.

Finally, while the study by Asami et al. does not involve radiomics, the authors explored different ML approaches to build predictive models for LNM assessment using clinical and MRI characteristics [43]. They found ML could be used to identify patients at high-risk for recurrence, irrespective of their pathological LNM status, potentially indicating the need for postoperative therapy. Interestingly, within the group of patients who did not exhibit pathological LNM, those who received a positive LNM prediction from this model experienced worse clinical outcomes than those with a negative LNM prediction. Given the model’s high specificity (up to 87% depending on the ML algorithm), it could represent an accurate tool to identify patients who may benefit from a lymphadenectomy. Table 2 presents a summary of the reviewed articles on the topic.

## 3. Prognostic Factors Related to Tumour Histology

### 3.1. Histological Subtypes and Tumour Grade

Histological tumour type plays a crucial role in predicting the prognosis of endometrial carcinoma, with the fifth edition of the WHO Classification of Tumors, Female Genital Tumors setting the standards for proper classification [44]. It recognizes several histological types, including: (1) endometrioid carcinoma (EEC), which can be further categorized as low grade (grades 1 and 2) or high grade (grade 3); (2) serous carcinoma (SC); (3) clear cell carcinoma (CCC); (4) mixed carcinoma (MC); (5) undifferentiated carcinoma (UC); (6) carcinosarcoma (CS); other uncommon types, such as (7) mesonephric-like and (8) gastrointestinal mucinous type carcinomas.

Under the FIGO staging system, updated in 2023, EC can be classified as non-aggressive histological type I, including low-grade (grades 1 and 2) endometrioid carcinomas (EECs), and aggressive histological type II, including high-grade EECs (grade 3) and non-endometrioid tumours (SC, CCC, MC, UC, CS, mesonephric-like tumours, and gastrointestinal-type mucinous carcinomas) [16].

Lefebvre et al. developed ML models based on radiomics for the non-invasive detection of high-risk histopathological characteristics in EC using multiparametric MRI, and specifically tumour grade [31]. The AUC was 0.74 in both train and test, demonstrating high stability, with the model including texture features from T2w, DWI, ADC and multiphase DCE sequences. This performance is higher compared to that reported for a different DCE (delayed phase only) model by Fasmer et al., showing an AUC value of 0.63 for grade 3 EC identification, suggesting that a multiparametric radiomic model might be better [24]. In the same study, an AUC of 0.73 for the identification of non-endometrioid EC was also reported.

Zhang et al. integrated ADC tumour values and the radiomic features (from T2w images) using dedicated nomograms to predict five EC risk factors and demonstrated a good performance in identifying the type and grade of EC, achieving AUCs of 0.851 and 0.867 for type and 0.959 and 0.880 for grade in the training and validation cohorts, respectively [45]. The authors did not perform a formal comparative analysis between the nomograms and the preoperative biopsy since the reports of the latter often did not include details on grade nor histological type due to the limited sampling. In this light, the authors speculate that their nomograms could complement biopsy especially in those cases. These authors also found that the ADC value was significantly lower in the type II EC compared to the type I EC group. Indeed, the type II EC includes poorly differentiated subtypes which often lack well-defined glandular structures and display flaky or papillary solid tumour tissue arranged densely [45].

Finally, Li et al.’s study presented a model based on whole-tumour T2w radiomics features that showed a good accuracy (F1-score of 0.72) in determining the histological subtype of EC [34].

### 3.2. Lymphovascular Invasion

Lymphovascular space invasion (LVSI) is characterized by the presence of tumour cells within spaces lined by endothelial cells outside the direct invasive border and can only be diagnosed on a histological basis. Specifically, LVSI in EC is defined as the infiltration of cancer into lymphatic and/or vascular spaces within the uterine myometrium [46].

LVSI has been recognized as a strong predictor of nodal involvement in EC and of high-risk recurrence and poor survival rate in early-stage EC [47]. As surgical staging decreases in importance, factors such as LVSI can serve as proxies for guiding future treatment strategies. The presence of LVSI, with its notable negative predictive value, aids in categorizing patients based on the risk of occult nodal disease. Even though MRI is widely used to assess EC and its local staging, LVSI cannot be evaluated by diagnostic imaging nor pre-surgery biopsy: its presence can only be verified after surgical resection, thus making the search for LSVI prediction tools particularly relevant.

Some authors proposed radiomics nomograms as a tool to assess the likelihood of a patient having a positive LVSI, potentially offering valuable assistance in clinical decision-making. Luo Y et al. created a nomogram algorithm that included a radiomics signature (obtained with radiomics features extracted from multiple sequences and 3D manual segmentations), age, and tumour grade, demonstrating effective predictive capabilities for preoperative LVSI in EC (AUCs 0.820 and 0.807 in the training and test cohorts, respectively) [48]. With a different approach, Zhang et al. realized a nomogram for LSVI prediction integrating radiomics features (T2w) together with ADC values (both from the EC lesion) with a similar AUC in the training dataset (0.82) but a significant performance drop in the test set (0.75) [45].

In a more recent study, Liu et al. combined radiomics features (multisequence, whole tumour) and clinical parameters (age and CA125 antigen) from a multicentre (five institutions) cohort (339 patients with early-stage EC) to create a model which resulted in a good performance with an AUC in the train and test sets, respectively, of 0.89 and 0.85 [49]. Once again, the authors embraced a nomogram as a tool to integrate radiomics and clinical data. It should, however, be noted that the performance of the combined nomogram was not significantly higher than that of the radiomics signature alone. The main characteristics of the studies investigating LSVI prediction with MRI radiomics can be found in Table 3.

## 4. Prognostic Factors Related to Tumour Genetics

Radiogenomics is an emerging field which explores the relationship between imaging features and underlying genetic characteristics in cancer. It plays a pivotal role in guiding treatment decisions by identifying imaging biomarkers linked to therapeutic responses and resistance [50]. This allows for the selection of targeted therapies that are more likely to be effective. Despite its promise, radiogenomics faces challenges such as standardization of imaging protocols, data sharing, and the need for large-scale validation studies. The integration of AI and ML algorithms is expected to enhance the efficiency and accuracy of radiogenomics analyses.

**Figure 1:**
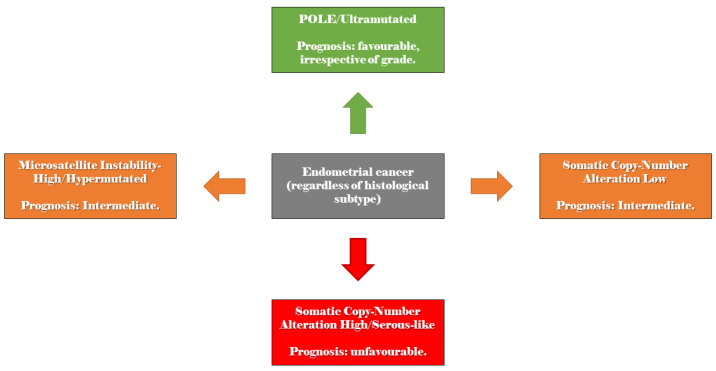
Schematisation of the endometrial cancer molecular classification proposed by The International Federation of Gynecology and Obstetrics, with a focus on prognosis. Note that prognosis is influenced by oestrogen receptor expression and histological grade for Somatic Copy-Number Alteration Low.

The International Federation of Gynecology and Obstetrics (FIGO) proposed a molecular classification processed by The Cancer Genome Atlas (TCGA) for EC [51], schematised with a focus on prognosis in Figure 1.

A simplified version of this molecular classification has been elaborated by Proactive Molecular Risk Classifier for Endometrial Cancer (ProMisE) using a combination of immunohistochemistry and mutation analysis and comprehends four genomics groups of EC [16]: (1) POLEmut with a favourable prognosis; (2) MMRd and (3) SMP with an intermediate prognosis; (4) p53abn with a poor prognosis.

Preoperative molecular assessment serves not only in stratifying risks but also aids in making customized decisions regarding surgery, determining the need for adjuvant therapy, and planning individualized follow-up strategies [52]. Due to these considerations, the updated guidelines from ESGO/ESTRO/ESP for managing EC patients have incorporated the ProMisE maintaining the classification into four risk classes (low, intermediate, high-intermediate, high) [53,54].

There are few research articles in the field of radiogenomics and EC, aimed at improving risk stratification in EC patients by collecting all the necessary information preoperatively and overcoming the high cost of molecular analysis.

In the study by Hoivik et al. on 866 EC patients, the integration of MRI with histologic, transcriptomic, and molecular biomarkers through radiogenomics was explored for accurate prognosis and tailored treatment [55]. A whole-tumour radiomic profiling was performed revealing clusters linked to high-risk histological features and poor survival. From the identified radiomic risk-groups, an 11-gene high-risk signature emerged, demonstrating prognostic significance in validation cohorts and alignment with The Cancer Genome Atlas (TCGA) molecular class associated with poor survival, particularly characterized by copy-number high/p53-altered status. The study suggests that MRI-based integrated radiogenomics profiling could refine tumour characterization, potentially contributing to improved prognostication and guiding future treatment approaches in EC.

Similarly, Celli et al. conducted a radiogenomic, retrospective and multicentric study including a more limited sample of 64 patients diagnosed with EC to separate low- from high-risk patients, based on the latest ESMO-ESGO-ESTRO guidelines [56]. A risk class model incorporated two radiomic features from ADC and T2W MRI sequences and resulted in an AUC of 0.74 for low-risk prediction. Additionally, another model for LVSI prediction was processed, using a single feature from ADC maps, reaching an AUC of 0.59. These models gave a medium-to-high diagnostic performance and showed how radiomics and radiogenomics analyses could provide a strong advantage in assessing EC risk stratification and reduce errors or partial information derived from routine MRI and biopsy preoperatively.

## 5. Overall Risk Stratification

Rather than looking separately at each single prognostic factor, more comprehensive approaches to the risk stratification of EC patients by means of radiomics/AI have been explored. A complete and reliable prediction model to identify low-risk or high-risk EC patients stratified on the basis of multiple tumour-specific characteristics would potentially pave the way for personalized management. It might be speculated that setting this goal would allow us to harvest the true potential of radiomics and diagnostic imaging AI, possibly unveiling biological correlates and imaging biomarkers that would remain concealed when focusing on isolated prognostic factors. On the other hand, the increased complexity of this prediction task and the possible bias to mitigate during model training (e.g., properly defining the classes) should also be considered. In this light, it could arguably be preferable to complement the radiologist work with prediction models built to assess those prognostic factors that it is not currently possible to evaluate with MRI.

Chen et al. proposed a radiomics T2-weighted model to stratify the risk of EC patients considering exclusively locally confined disease (pT1a or pT1b) and the following definition of low-risk patients (pT1a and either G1 or G2 endometrioid EC) [57]. Their model demonstrated an AUC of 0.815 (95% confidence interval: 0.588–1) in the validation cohort for differentiating high and low risk groups. This finding underscores the potential of T2-weighted radiomics applications for EC but necessitates cautious interpretation. Notably, with the lack of external independent validation as well as of feature stability testing for multiple segmentations and a wide confidence interval, the performance claim of this feasibility study might be overly optimistic. Conversely, in a recent study with a significantly larger multicentre cohort (717 vs. 102), a radiomics signature was developed for pre-treatment identification of high-risk EC (defined as per the ESMO guidelines) [58]. Validation on two independent and external datasets yielded noteworthy outcomes (AUCs of 0.75 and 0.85 in validation groups 1 and 2, respectively) [59]. However, their model requires data from three different MRI sequences (i.e., T2-weighted, diffusion-weighted, and contrast-enhanced images). While these sequences are all usually acquired for MRI staging of EC, the enhanced performance comes at the expense of heightened complexity, an increased and time-consuming segmentation workload, and potential limited applicability, especially for patients with contraindications to contrast medium administration. Notably, Yan and colleagues found, in their study, that T2-weighted features held the highest weight in the radiomics signature after feature selection. This latter finding was confirmed from a different research group with a subsequent study in which a T2-weighted model was trained and externally validated to identify low-risk patients (pT1a and either G1 or G2 endometrioid EC) [60]. While the model’s performance was lower (AUC of 0.71 and 0.72 in the train and validation sets, respectively), it resulted stable at independent external validation, demonstrating a good potential for generalizability. In a more recent work, Miccò et al. adopted a similar definition of low risk and their T2-weighted-based model showed a better overall performance (AUC train/validation of 0.84/0.76) [32]. The observed performance drop from train to validation can be partly explained by the relatively high number of included features (*n* = 15) compared to the number of enrolled patients (*n* = 96), which increases the risk of overfitting. Finally, it is worth to mention another study on a larger training population (*n* = 413) with external validation (*n* = 82) showing interesting results for the identification of high-risk patients (all but pT1a and either G1 or G2 endometrioid EC) [34]. In this case, the AUC for the classification model based once again on T2-weighted sequences was 0.84 (95% CI 0.75, 0.91), suggesting that larger datasets are probably required to train higher performance models.

## 6. Still More Challenges than Opportunities

### 6.1. Beyond Feasibility: Clinical Applicability and Usefulness

In light of the evidence summarized above, it can be stated that these novel quantitative strategies to analyse medical images (namely hand-crafted radiomics and DL approaches) might find potential applications to MR images for patients affected by EC, at least on a technical level (Table 4).

However, while the core of the relevant literature has significantly expanded in the last few years, research appears to be substantially stalled at the proof-of-concept and feasibility stage, with most (if not all) of the previously identified challenges still to be properly faced [61]. It is undeniable that such advanced strategies add a layer of complexity with the potential to further increase the already crushing workload burden for radiologists. For instance, manual tumour segmentation, especially when 3D annotations on multiple sequences are required to power the predictive model, represents a notably time-consuming and tedious task. A possible solution is represented by implementing automated segmentation strategies, as proposed by Kurata and colleagues [62]. It should also be considered that, contrary to radiomics, DL solutions often do not require image segmentation. Nonetheless, from significant complexity arise additional issues, such as the difficulty in understanding the intricacies of the decision-making mechanism employed by the model. To gain full trust from patients, clinicians, and surgeons in these tools, we cannot refrain from demonstrating that their clinical value is built on the foundation of robust correlations between imaging data and the physiopathology of endometrial tumours, and that potential sources of bias have been addressed [63].

With respect to clinical usefulness, it could be argued that every new tool should be evaluated within the context of the current standards and its value should be realistically demonstrated in terms of integration rather than substitution [64]. Establishing this incremental value requires, among others, cost-benefit analyses, holistic models (integrating imaging, clinical and radiomics data), as well as direct comparisons with valid alternatives (e.g., sentinel lymph node biopsy for nodal assessment). It also interesting to note that most of the reviewed studies propose the clinical implementation of their AI/radiomics models by means of a nomogram. However, the suitability of these static graphics to represent dynamic models such as those generated by ML has been questioned and many consider them to be obsolete [30]. To bring these technologies to the next step, all the above-mentioned shortcomings need to be properly addressed.

By changing the perspective, it is also not necessarily the case that just because an approach can work, it is then inherently useful [65]. One cannot disregard how previous promising quantitative MRI analyses approaches for the risk stratification of EC patients have not effectively translated into clinical practice. This is, for example, the case of quantitative diffusion and perfusion parameters, including several imaging biomarkers such as ADC values and Ktrans that have been vastly investigated before the AI emergence in radiology [66,67,68]. These parameters can be obtained with greater ease and are far better explainable in terms of biological significance and correlates compared to radiomics features and DL predictions. Should we, as researchers, follow the hopes and hypes of these newer tools or should we rather keep focusing our efforts on those already more established and familiar imaging biomarkers to finally foster their clinical adoption?

### 6.2. Reproducibility, Generalizability and Data Openness

The ongoing reproducibility crisis, which is afflicting every field of scientific research, poses a concerning and recognized issue for AI in radiology, with data leakage representing one of the main underlaying causes [69,70]. In brief, data leakage occurs when the model makes spurious connections between features and prediction object due to pitfalls in data collection, sampling, or pre-processing strategies (e.g., using the Synthetic Minority Oversampling Technique to address class imbalance before splitting a dataset into training and testing datasets) [71]. This typically leads to an overestimation of the model’s performance which subsequently drops when tested on external independent data (i.e., poor generalization of results). However, the roots of AI’s reproducibility crisis in imaging informatics run even deeper as most studies cannot be replicated to verify reproducibility of results due to the lack of data openness [72]. Regarding AI in gynaecologic imaging, a recent systematic review of the literature confirmed that this field represents by no means an exception with generalizability concerns (e.g., performance drop when applying models trained on highly curated datasets to real-life scans affected by frequent artifacts) and reproducibility issues (e.g., model and/or code and/or data not openly shared) [73]. Without a widespread adoption of open science practices, researchers might not be able to make significant advancements in the field of AI and radiomics in radiology. Another underlaying cause behind poor generalizability/reproducibility can be tracked back to a weak spot in handcrafted radiomics pipelines, namely image segmentation [71]. Specifically, not all radiomics features are robust to repeated segmentation and feature stability to annotations performed by different operators or using segmentation perturbation should be evaluated to exclude unstable features and build more reliable models [74]. To address the radiomics workflow’s complexity and its challenges in reproducibility, the CheckList for EvaluAtion of Radiomics research was recently published and offers a valuable unified documentation standard describing minimum requirements [75].

### 6.3. Commercially Available Solutions

Ideally, the final aim of scientific investigation on radiomics and DL should overall be to enable to creation of certified and reliable software cleared for medical use and capable of improving patient outcomes. Indeed, commercially available products for bringing AI in radiology are overall increasing in number, but these are often supported by low methodological quality studies and limited evidence of clinical benefit [76,77]. Possibly as a consequence of what discussed in this section, and to the best of our knowledge, there are currently no commercially available solutions at present [78]. However, even if this was not the case, we would recommend considering with great caution the idea of purchasing such a product in the near future [79]. Among the plethora of questions software manufacturers should be required to provide satisfactory answers to regarding their products, we would like to mention the following: what strategies are being implemented to minimize model’s performance decay overtime? Due to the ever-evolving nature of the clinical setting (e.g., tumour classification updates, time-related changes in target population, data acquisition), differences might emerge between the data used for model training and that collected during practice, a phenomenon known as data drift which if not mitigated unavoidably leads to performance loss [80]. Retraining the model represents a possible solution, but the whole process requires time as well as expenses and the product would then intuitively necessitate a new certification. Overall, radiomics and AI models can only be as good as the datasets they are built on (garbage-in, garbage-out), which further emphasizes the paramount importance of developing and validating these models using diverse and representative datasets [71].

Despite all the issues mentioned, it deserves to be acknowledged that radiology is a leading subspecialty for AI utilization in medicine, with the vast majority of FDA-approved medical devices belonging to the field [81]. In this light, hope might be placed in the prospect that further and more significant progress could be achieved in a not-so-distant future.

## 7. Conclusions

In the quest to enhance MRI’s role in EC assessment, AI and radiomics offer promising strides. Yet, amidst encouraging results, the field grapples with heterogeneous data, methodological gaps, and a scarcity of validation studies. The road to reproducibility and clinical integration remains challenging, prompting the scientific community to engage in a thoughtful consideration on the path forward.

## Figures and Tables

**Table 1:** Main characteristics of the radiomic model for DMI prediction studies.

Study	Design	Population (Train/Validation)	MRI Scanner and Sequences	Segmentation	Modelling	Performance (AUCt/AUCv)
Dong et al. (2020) [25]	Retrospective	24/48	1.5 T and 3 T T2w, DCE	2D, automatic	DL	NR
Chen et al. (2020) [26]	Retrospective	392 */138	1.5 T T2w	3D, automatic and manual	DL	0.85/0.78
Fasmer et al. (2020) [24]	Retrospective	108/30	1.5 T and 3 T DCE	2D and 3D, manual	Statistic	0.84/0.76
Stanzione et al. (2020) [23]	Retrospective	43/11	3 T T2w	2D, manual	ML	0.92/0.94
Han et al. (2021) [27]	Retrospective	163/NA	3 T T2w, DWI	3D, manual	ML	0.85
Zhao et al. (2022) [29]	Retrospective	107/56	1.5 T and 3 T T2w, DCE	3D, manual	ML	0.94/0.87
Lefebvre et al. (2022) [31]	Retrospective	94/63	1.5 T T2w, DWI, DCE	3D, manual	ML	0.86/0.81
Otani et al. (2022) [33]	Retrospective	150/50	3.0 T and 1.5 T T2w, DWI, DCE	3D, manual	ML	0.76/0.83
Miccò et al. (2022) [32]	Retrospective	73/23	1.5 T T2w	3D, manual	ML	0.85/0.68
Li et al. (2023) [34]	Retrospective	292/60	1.5 T and 3 T T2w	3D, manual	ML	NR/0.79

* A subset of the train dataset was employed for internal validation. DL: deep learning; ML: machine learning; AUC: area under the curve; AUCt: area under the curve in the training test; AUCv: area under the curve in the validation test; MRI: magnetic resonance imaging; DWI: diffusion weighted imaging; DCE: dynamic contrast-enhanced; NA: not applicable; NR: not reported.

**Table 2:** Main characteristics of radiomics studies for lymph node metastasis prediction in endometrial cancer.

Study	Design	Population (Train/Validation)	MRI Scanner and Sequences	Segmentation	Modelling	Performance (AUCt/AUCv)
Xu et al. (2019) [39]	Retrospective	140/60	3 T T2w, DCE	3D, manual	ML	0.89/0.88
Fasmer et al. (2020) [24]	Retrospective	108/30	1.5 T and 3 T DCE	2D and 3D, manual	Statistic	0.73/0.72
Yang et al. (2022) [42]	Prospective	165/71	3 T ADC	3D, manual and semi-automated	ML	NR/0.89
Otani et al. (2022) [33]	Retrospective	150/50	3.0 T and 1.5 T T2w, DWI, DCE	3D, manual	ML	0.80/0.72
Liu et al. (2022) [40]	Retrospective	350/354	1.5 T T2w, DWI, ADC, DCE	3D, manual	ML	0.85/0.83
Bo et al. (2022) [41]	Retrospective	95/41	3 T T2w, DWI, DCE	3D, manual	ML	0.94/0.92

ML: machine learning; AUC: area under the curve; AUCt: area under the curve in the training test; AUCv: area under the curve in the validation test; MRI: magnetic resonance imaging; DWI: diffusion weighted imaging; ADC: apparent diffusion coefficient; DCE: dynamic contrast-enhanced; NR: not reported.

**Table 3:** Main characteristics of radiomics studies for lymphovascular space invasion prediction.

Study	Design	Population (Train/Validation)	MRI Scanner and Sequences	Segmentation	Modelling	Performance (AUCt/AUCv)
Luo et al. (2020) [48]	Retrospective	101/43	1.5 T and 3 T T2w, ADC, DCE	3D, automatic	ML	0.82/0.81
Zhang et al. (2021) [45]	Retrospective	120/90	3 T T2w	3D, manual	Statistic	0.82/0.75
Liu et al. (2022) [49]	Retrospective	350/354	1.5 T T2w, DWI, ADC, DCE	3D, manual	ML	
Lefebvre et al. (2022) [31]	Retrospective	94/63	1.5 T T2w, DWI, DCE	3D, manual	ML	0.86/0.80
Otani et al. (2022) [33]	Retrospective	150/50	3.0 T and 1.5 T T2w, DWI, DCE	3D, manual	ML	0.71/0.81
Miccò et al. (2022) [32]	Retrospective	73/23	1.5 T T2w	3D, manual	ML	0.92/0.81

ML: machine learning; AUC: area under the curve; AUCt: area under the curve in the training test; AUCv: area under the curve in the validation test; MRI: magnetic resonance imaging; DWI: diffusion weighted imaging; ADC: apparent diffusion coefficient; DCE: dynamic contrast-enhanced; NR: not reported.

**Table 4:** Potential MRI radiomics and artificial intelligence applications for the evaluation of endometrial cancer.

	Prognostic Factors Related to Tumour Stage	Prognostic Factors Related to Tumour Histology	Prognostic Factors Related to Tumour Genetics	Overall Risk Stratification
**Specific tasks**	DMI and LNMs	Tumour type and grade, LSVI	Molecular classification	Risk stratification
**Rationale**	Overcoming MRI limitations including limited accuracy, need of experienced readers and inter-observer variability	Enhancing the role of MRI in the evaluation of tumour characteristics that cannot be assessed with imaging	Fostering the widespread adoption of the novel risk stratification approaches	Providing comprehensive and patient-specific analysis to facilitate the transition towards personalized care

DMI: deep myometrial invasion; LNMs: lymph node metastases; LSVI: lymphovascular invasion.
